# ZnO–CuO nanocomposites for sustainable water treatment linking photocatalytic efficiency with ecotoxicological safety

**DOI:** 10.1038/s41598-026-53364-z

**Published:** 2026-05-25

**Authors:** Irina Zgura, Monica Enculescu, Nicoleta Preda, Liviu Nedelcu, Oana Rasoga, Valentin-Adrian Maraloiu, Catalin Negrila, Madalina Cercel, Monica Dinescu, Bogdan Ciprian Mitrea, Tom Matei Iacob, Cornelia Diac, Bogdan Bita, Cornelia Nichita, Judit Háhn, Zsolt Csenki-Bakos, Gergő Tóth, Balázs Göbölös, Illés Bock, Florian Littorie, Pascale Gillon, Marcela-Elisabeta Barbinta-Patrascu

**Affiliations:** 1 National Institute of Materials Physics, Atomistilor 405A, 077125 Magurele, Romania; 2 ICUB, Faculty of Physics, CTT-3Nano-SAE Research Centre, University of Bucharest, MG-38, 405 Atomistilor Street, 077125 Magurele, Romania; 3 Department of Electricity, Solid-State Physics and Biophysics, Faculty of Physics, University of Bucharest, 405 Atomistilor, 077125 Magurele, Romania; 4 Optospintronics Department, National Institute of Research and Development for Optoelectronics-INOE 2000, 409 Atomistilor, 077125 Magurele, Romania; 5 National Institute for Chemical – Pharmaceutical Research and Development, 112 Vitan Avenue, 031299 Bucharest, Romania; 6 Institute of Aquaculture and Environmental Safety, Hungarian University of Agriculture and Life Sciences, Páter Károly u. 1., Gödöllő, 2100 Hungary; 7 GEPEA, UMR 6144, CNRS, Oniris, IMT Atlantique, Nantes Université, 44600 Saint Nazaire, France

**Keywords:** p–n heterojunction, Rhodamine B degradation, Nanoparticle dissolution, Metal ion release, Multi-trophic ecotoxicity, Biotechnology, Environmental sciences, Nanoscience and technology

## Abstract

The development of sustainable water treatment technologies requires a balance between high pollutant removal efficiency and environmental safety across multiple trophic levels. In this study, ZnO–CuO nanocomposites were prepared by mechanical mixing of commercially available ZnO and CuO nanopowders, providing a scalable route. Structural and optical analyses confirmed the formation of p–n heterojunctions, enhancing charge separation and photocatalytic performance. Photocatalytic activity was assessed via Rhodamine B degradation under simulated solar irradiation, with ZnO97%–CuO3% showing the highest efficiency. To assess sustainability beyond contaminant removal, ecotoxicological assays were performed across multiple trophic levels, including a long-term 7-day assay on microalgae (*Scenedesmus obliquus*) and short-term tests involving plants (*Ocimum basilicum*), bacteria (*Aliivibrio fischeri*, Microtox®, 30 min), crustaceans (*Daphnia magna*, 48 h immobilization), and fish embryos (*Danio rerio*, up to 120 h post-fertilization). No adverse plant physiological effects were observed after exposure to the nanocomposite or treated water, unlike untreated dye solutions, which induced significant toxicity. Aquatic bioassays revealed organism-specific responses linked to particle-associated effects. Quantification of metal ion release and nanoparticle dissolution in aqueous matrices enabled differentiation between particulate and ion-mediated toxicity. Overall, the findings demonstrate that efficient photocatalytic water treatment can be achieved while maintaining a favorable ecotoxicological profile.

**Supplementary Information:**

The online version contains supplementary material available at 10.1038/s41598-026-53364-z.

## Introduction

Contamination of water resources with persistent organic pollutants including synthetic dyes, pharmaceutical residues and industrial effluents represents one of the most pressing environmental challenges worldwide. Owing to their chemical stability and resistance to natural degradation processes, these contaminants can persist for long periods in aquatic ecosystems leading to bioaccumulation and adverse effects on living organisms and trophic chains. Consequently, the development of efficient, sustainable and economically viable technologies for wastewater treatment remains a major priority.

In response to this challenge, a wide range of remediation strategies have been explored including biological approaches based on waste valorization in “green” bioremediation systems^1^ and advanced oxidation processes employing nanocomposite photocatalytic materials^2^. Among the various approaches investigated for pollutant removal, that based on heterogeneous photocatalysis driven by solar irradiation has emerged as a promising, environmentally friendly, clean and energy-efficient method for the degradation of toxic chemicals^3^. Hence, nanostructured metal oxide semiconductors have attracted considerable attention in recent decades for applications in environmental remediation^4^, catalysis^5^, and optoelectronics^6,7^ due to their favorable physicochemical properties, low cost, high stability and tunable properties^8^.

Zinc oxide (ZnO) and copper oxide (CuO) are among the most widely investigated metal oxide photocatalysts. ZnO is a wide-bandgap n-type semiconductor (~ 3.3 eV) with a hexagonal wurtzite structure, high exciton binding energy (~ 60 meV), and strong UV-light absorption, which makes it effective for the photocatalytic degradation of organic pollutants^9,10^ while CuO is a p-type semiconductor with a narrower bandgap (~ 1.2–1.9 eV), enabling visible light absorption and exhibiting notable photocatalytic and antimicrobial activity^11^.

The combination of ZnO and CuO into p–n heterojunction nanocomposites has been shown to enhance visible-light-driven photocatalysis by improving charge separation and reducing electron–hole recombination^12^. Several studies have reported improved photocatalytic performance of ZnO–CuO heterostructures compared to the individual components. For example, Alsulmi et al. demonstrated enhanced charge separation and higher degradation rates of organic dyes in ZnO–CuO systems^13^. Similarly, Messaid and co-workers developed polymer-based nanocomposites incorporating ZnO and Cu₂O nanoparticles for achieving high photocatalytic efficiency, particularly at elevated ZnO contents^14^. Despite these advances, the majority of reported studies remain predominantly focused on photocatalytic efficiency often overlooking critical aspects related to material synthesis complexity and ecological safety^9^, which involve high costs, multiple processing steps and limited scalability for industrial applications. In contrast, the direct mechanical mixing of commercial nanopowders offers a simple, low-cost and scalable alternative, yet this approach remains insufficiently explored in the literature^15^. In parallel, ecological safety assessments of photocatalytic nanomaterials are frequently restricted to a single model organism or are entirely absent, making realistic evaluation of environmental risks challenging. In addition to their photocatalytic function, the eco-toxicological profile of these nanocomposites is critical. Nanomaterials can interact with biological systems influencing enzymatic activity, generating oxidative stress, or impacting plant physiology—a concern explored in limited studies^16^.

Under environmentally relevant conditions, ZnO and CuO nanoparticles can partially dissolve, releasing Zn^2+^ and Cu^2+^ ions, with their release strongly influenced by water chemistry, including pH, ionic strength, and the presence of organic or inorganic ligands^17,18^. Dissolution can be substantial in complex biological or nutrient-rich media with reported values reaching 33–37% for ZnO and 9–18% for CuO (w/w) in cell culture media^19^. Similarly, studies in natural waters have shown that dissolved organic carbon and ionic strength strongly modulate zinc release and speciation^20^. These studies demonstrate that nanoparticle dissolution is highly matrix-dependent and that the environmental performance of metal oxide nanoparticles (MO-NPs) cannot be assessed independently of the surrounding aqueous medium. Given the toxicity of dissolved Zn^2+^ and Cu^2+^ toward aquatic organisms^18,21^, strategies to mitigate metal ion release at the source are increasingly being explored. Among them, modifying nanoparticle composition has emerged as a promising approach. In particular, the incorporation of small amounts of CuO into ZnO-based materials has been reported to also limit copper ion release (D. Singh & Kumar)^22^.

Terrestrial plants need the presence of copper and zinc for their physiological processes (e.g., plant growth, activation of some enzymes, respiration and photosynthesis). These essential micronutrients (ions or nanoparticles) may have phytotoxic effects at high doses but their simultaneous addition can mitigate their toxicity^23,24^. The study of Adamczyk et al. revealed that the addition of copper or zinc to the soil increased the content of bioactives in common nettle and basil^24^. In our study, we chose the basil plant (*Ocimum basilicum* L.) because (i) it is a condiment and a medicinal plant recognized for its therapeutic properties^25^, and (ii) it is an aromatic herb highly sensitive to soil^26^. Complex aquatic ecotoxicity assessments of engineered nanomaterials typically encompass multiple trophic levels to capture a realistic spectrum of biological responses in natural ecosystems. Standard ecotoxicological frameworks recommend testing organisms at the base of the food web (microbial and primary producers such as *Vibrio fischeri* or algae), intermediate consumers (e.g., crustaceans such as *Daphnia magna*), and higher trophic representatives (fish species such as *Danio rerio*) to derive comprehensive hazard profiles and regulatory endpoints (e.g., EC₅₀/LC₅₀ values, where EC₅₀ is effective concentration and LC₅₀ is lethal concentration, both concentrations being required to cause a specific effect in 50% of a test population). Such three-level testing strategies have been widely applied in the literature demonstrating that ecotoxicological responses vary markedly across trophic categories being strongly influenced by nanoparticle physicochemical properties as well as dissolution behavior in aquatic media^27^. For instance, median toxicity values reported for ZnO and CuO nanoparticles indicate differential sensitivity among organisms, with crustaceans and algae often exhibiting higher susceptibility than bacteria in acute exposure assays underscoring the importance of multi-level assessments for robust environmental risk evaluation^27^. Moreover, comprehensive reviews covering a broad range of nanomaterials (including metal oxide nanoparticles) consistently emphasize that evaluating effects across diverse organism types from microbes and primary producers to invertebrates and vertebrates is essential for understanding potential impacts on aquatic ecosystem structure and function^28,29^. In this context, an integrated approach that combines simplicity of fabrication, photocatalytic performance and rigorous ecological safety assessment is needed.

In the present study, ZnO–CuO nanocomposites obtained by mechanical mixing of commercially available metal oxide nanopowders were thoroughly evaluated from structural, morphological, optical, photocatalytic and ecotoxicological perspectives. The dissolution behavior of ZnO–CuO nanocomposites was examined in two aqueous matrices: deionized water and a microalgae culture medium, Bold’s Basal Medium (BBM). The biological impact was investigated at multiple trophic levels. Plant physiological responses including photosynthesis and respiration were assessed using *Ocimum basilicum* L. The physiological responses of the freshwater microalga *Scenedesmus obliquus* in response to the direct and indirect stress induced by the metal oxide nanopowders were also quantified. The aquatic ecotoxicity of the metal oxide nanopowders and the detoxification efficiency of the treated water were assessed on three trophic levels using a prokaryote (*Aliivibrio fischeri*), an invertebrate (*Daphnia magna*) and a vertebrate (*Danio rerio*) test organism. By correlating material properties with photocatalytic efficiency and biological responses across plant and aquatic organisms, this work demonstrates that efficient pollutant degradation must be assessed simultaneously with the ecological impact of both employed materials and treated water. The results show that ZnO–CuO nanocomposites prepared via a simple mechanical mixing approach can achieve effective photocatalytic degradation of organic pollutants while maintaining a favorable ecotoxicological profile, offering valuable insights for the development of sustainable water purification technologies.

## Materials and methods

### Materials and nanocomposite preparation

Commercial metal oxide nanopowders purchased from Sigma-Aldrich (Zinc oxide nanopowder with particle size ≤ 100 nm and Copper(II) oxide nanopowder with particle size < 50 nm) were used to prepare both mono-component (ZnO and CuO) and composite (ZnO–CuO) samples. The investigated samples were homogenized by mechanically mixing metal oxide powders (ZnO, CuO, and ZnO–CuO nanocomposites in various mass ratios) in an agate mortar for 15 min. The composition was controlled by precise weighing (to five decimal places) of the individual components. The powder samples were labeled as follows: ZnO100% NPs, ZnO99%–CuO1% NPs, ZnO97%–CuO3% NPs, ZnO95%–CuO5% NPs, ZnO90%–CuO10% NPs and CuO100% NPs.

### Structural and physicochemical methods

Structural characterization was performed by X-ray diffraction (XRD) using a XRDynamic 500 diffractometer (Anton Paar), which employs CuKα radiation (λ = 0.154 nm). A Ni/C multilayer mirror was used to filter CuKβ radiation and the instrument was equipped with a Pixos 2000 1D detector featuring 256 independent acquisition channels. The diffraction patterns were recorded at room temperature using the Bragg–Brentano geometry over a 2θ range from 10° to 120°, with a step size of 0.02° and an acquisition time of 0.5 s per step. To verify proper alignment of the diffractometer, the NIST SRM 1976c (corundum) standard was used. Morphological analysis was carried out using a Carl Zeiss (Oberkochen, Germany) Gemini 500 field emission scanning electron microscope (FESEM) working in high vacuum (HV), from 0.2 to 30 kV, and equipped with a LaB6 emitter, InLens, and SE2 detectors. The elemental composition of the samples was evaluated using a Bruker QUANTAX 200 energy-dispersive X-ray spectrometer detector (EDS) with Peltier cooling and an energy resolution < 129 eV at Mn-Ka. The optical properties were investigated using diffuse reflectance spectroscopy and Fourier-transform infrared spectroscopy (FTIR). Reflectance and FTIR spectra were recorded using a Perkin-Elmer Lambda 45 spectrophotometer equipped with an integrating sphere and a Jasco 6800 spectrometer, respectively. The photoluminescence (PL) spectra were acquired using a FL 920 Edinburg Instruments spectrometer with a 450W Xe lamp excitation and double monochromators on both excitation and emission. Particle Size Distribution DLS was performed using the Dynamic Light Scattering (DLS) technique with a Zetasizer Nano ZS instrument (Malvern Instruments Inc.). Transmission Electron Microscopy (TEM) investigations were carried out using an aberration probe-corrected JEOL ARM200F microscope operated at an acceleration voltage of 200 kV and equipped with a JEOL JED-2300 T unit for Energy- Dispersive X-ray (EDX). Images were acquired using a Gatan Orius CCD camera at magnification from 40000 × to 2000000x. EDS mapping in STEM mode was also performed in order to find a heterojunction in ZnO97%–CuO3% NPs. Powders were dispersed in ethanol and then they were sonicated for 5 min. A 5 μL drop of each suspension was deposited on Au grid covered with lacey carbon film and let to dry at room temperature. X-ray Photoelectron Spectroscopy (XPS) measurements were performed in a SPECS Surface Multi Analysis System based on a PHOIBOS 150 electron analyzer. A monochromatic X-Ray source with Al anode (Al Kα radiation with 1486.61 eV) was used for samples excitation while a flood gun of FG15/40 type provided the necessary electron flow for charge compensation. Three samples were investigated: ZnO100% NPs, CuO100% NPs and ZnO97%–CuO3% NPs. The fitting of the spectra was performed in a Spectral Data Processor Software using Voigt functions and usual relative sensitivity factors.

### Photocatalytic experiments

The synthetic wastewater was prepared by dissolving one of the most commonly used dyes, Rhodamine B (RhB, Sigma Aldrich) in double-distilled water. Irradiation was performed using a solar simulator with a narrow collimated beam, SF300-A (Sciencetech, London, ON, Canada), equipped with an Air Mass AM1.5G filter (spot size: 25 mm diameter, irradiance: 1 Sun) and an integrated electric shutter with controller, coupled to a 300 W xenon arc lamp. In the photocatalysis experiments, an amount of 1 mg of photocatalyst powders based on ZnO, CuO or ZnO–CuO nanocomposites in various compositions were added to 1 mL of the aqueous RhB solution (10⁻^5^ M). The UV–Vis spectra of the samples were recorded in the 400–650 nm range using a CARY 5000 UV–Vis-NIR spectrophotometer for monitoring the absorption maximum of RhB located at 554 nm. The degradation of the dye was performed under visible light irradiation for a period of 60 min. Before irradiation, the catalyst–dye suspension was magnetically stirred in the dark for 15 min to establish adsorption–desorption equilibrium between Rhodamine B molecules and the photocatalyst surface. A control experiment was performed under dark conditions to achieve the adsorption–desorption equilibrium between Rhodamine B molecules and the catalyst surface. At regular irradiation intervals, the sample was withdrawn and the absorption spectrum was collected.

The degradation of RhB in the absence or in the presence of photocatalyst powder was determined by tracking the change in absorbance at 554 nm, the photodegradation efficiency being calculated using the following equation:

$$Degradation\; efficiency \left( \% \right) = \frac{{C_{0} - C}}{{C_{0} }} \times 100$$

where: ${C}_{0}$ is the initial dye concentration, *C* is the dye concentration at the time *t*.

The synthetic wastewater samples containing Rhodamine B (10⁻^5^ M) were labeled according to their treatment conditions. The sample containing RhB and the ZnO97%–CuO3% nanocomposite prior to irradiation was denoted as WRhB ZnO97%–CuO3% NPs, while the corresponding sample after the photocatalytic degradation process was denoted as TW RhB ZnO97%–CuO3% NPs.

### Nanoparticle dissolution experiments

The dissolution of metal oxide nanoparticles (MO-NPs) and its dependence on irradiation and water chemistry was investigated using a two-step experimental design. In both steps, metals were quantified by separating dissolved and particulate fractions using the improved methodological approach, ultrafiltration/ Inductively Coupled Plasma–Atomic Emission Spectrometry (UF/ICP-AES) adapted from previously described methodologies, as described below^30,31^. Thus, first, dissolution kinetic of the ZnO–CuO composite with the best photocatalytic activity was conducted in deionized water. Experiments were performed at a nanoparticle concentration of 100 mg/L in 100 mL of solution using 200 mL borosilicate glass beakers (Duran). The pH was adjusted to 7.3 prior to nanoparticle addition and suspensions were continuously stirred at room temperature. Two conditions were tested: dark and UV irradiation (365 nm, 92 mW·cm⁻^2^ measured at the inner wall of the beakers). Dissolution was monitored over 3 h, with samples collected at selected time points. All experiments were conducted in triplicate. In a second step, dissolution experiments were performed to compare nanoparticle behavior in deionized water and in a microalgal culture medium. These experiments were carried out in the dark under identical conditions (100 mg/L, 100 mL, pH 7.3, continuous stirring, room temperature) using a fixed contact time of 30 min, allowing direct comparison between matrices. Each condition was tested in triplicate. At the beginning (T_0_) and at the end of each experiment, 15 mL aliquots were collected and centrifuged at 5000 *g* for 45 min to sediment nanoparticles and precipitates. Supernatants were recovered and ultrafiltered using Amicon Ultra-15 centrifugal units equipped with 3 kDa Ultracel membranes (Merck Millipore) for 60 min at 3000 *g*. Permeates were immediately acidified to 5% (v/v) HNO_3_ (VWR) prior to analysis and were assumed to contain predominantly dissolved Zn^2+^ and Cu^2+^ ions while larger particles and complexes (> ~ 1.3 nm) were retained^32^. Pellets were digested separately with 2 mL of concentrated HNO_3_ (69%) at 90 °C for 3 h to ensure complete dissolution of metal oxides. After evaporation of nitric acid at 120 °C, residues were diluted in 5% HNO_3_. Zinc and copper concentrations were quantified by ICP-AES (iCAP 6300 Radial, Thermo Scientific). Calibration curves were prepared using certified standards (1000 ppm, Chem-Lab) in the ranges 0.2–10 mg Zn^2+^/L and 0.1–4 mg Cu^2+^/L. Analyses were performed in triplicate and included procedural blanks, spiked matrix blanks, and scandium as an internal standard.

### Ecotoxicological assays

#### Plant physiological assays (*Ocimum basilicum*)

The experimental evaluation of the photosynthesis and respiration processes in plants was conducted in a controlled laboratory greenhouse equipped with automated illumination and irrigation systems to maintain optimal growth conditions for *Ocimum basilicum L.* plants (Ilfov 44°21′N 25°58′E). The plants were treated daily (for 72 h, at 24-h intervals) with aqueous suspensions (1 g/L) containing metal oxide powders: ZnO100% NPs, CuO100% NPs and ZnO97%–CuO3% NPs. Each plant received 20 mL/24 h, applied directly at the root level. The control group was treated identically using only distilled water. After a treatment period, the plants were placed in a custom-built bioclimatic photosynthesis chamber, where CO₂ concentration was measured in real-time using a Smart CO₂ Sensor (Data Harvest, UK). This device detects CO₂ level variations in ppm during photosynthesis and plant respiration processes. To ensure accurate and synchronized data acquisition, the sensor was connected to the EasySense2 software platform (Data Harvest, UK), which facilitated real-time visualization, calibration and export of CO₂ concentration data. The software is available at: https://store.data-harvest.co.uk/easysense2.

#### Microalgae assays (*Scenedesmus obliquus*)

The culture medium used was Bold Basal Medium (BBM) [UTEX 393 from Culture Collection of Algae (University of Texas at Austin, USA; https://utex.org/products/utex-0393). Bold’s Basal Medium (BBM) n.d. https://utex.org/products/bolds-basal-medium-bbm (accessed July 23, 2024).] prepared in autoclaved deionized water (MilliQ, HX 7040 SD) filtered at 0.2 µm. The medium was supplemented with 1.29 g/L of sodium bicarbonate to promote microalgal growth. The final pH of the culture medium was adjusted to 7.3 with a 1 M sodium hydroxide solution.

The microalga used was *Scenedesmus obliquus* (UTEX 393). The exponential phase of this strain was maintained in 500 mL glass Erlenmeyer flasks containing 250 mL of the appropriate medium. Stock cultures were carried out under constant artificial light, at an LED intensity of 80 µmol photons·m⁻^2^ s⁻^1^, at a temperature of 22 ± 1 °C, in static conditions including a manual agitation 3 times per day.

Toxicity assays were performed using *Scenedesmus obliquus* to assess the effects of metal oxide nanoparticles and of dissolved metal ions released from these nanoparticles. Experiments were conducted in two successive steps in order to distinguish nanoparticle-related effects from ion-mediated toxicity. In a first set of assays, microalgal cultures were exposed to the ZnO–CuO nanocomposite with the best photocatalytic activity at a final concentration of 100 mg/L. Nanoparticles were added from stock suspensions (20 g/L ZnO and 5 g/L CuO) after vortex mixing and sonication for 10 min at 45 kHz in an ultrasonic bath (VWR, USC100T). The suspensions were then introduced into the corresponding algal culture media. Cultures were incubated in duplicate under standard growth conditions as described above. In a second set of assays, microalgae were exposed to dissolved zinc and copper ions at concentrations representative of those measured during nanoparticle dissolution experiments. Ionic solutions were prepared from metal sulphate salts (Sigma-Aldrich, USA) to match the Zn^2^⁺ and Cu^2^⁺ concentrations quantified in the 3 kDa permeates obtained during dissolution tests. These assays were performed in triplicate, using the same culture media and incubation conditions as for the nanoparticle exposures. Control cultures without nanoparticles or added metal ions were included in all experiments to allow comparison with exposed conditions.

To monitor microalgae growth daily during the 7-day cultivation, spectrophotometric measurements at 750 nm were performed for each replicate using an UV–visible spectrophotometer (Jasco, V650). Each measurement was performed in triplicate after vortexing the samples to prevent cell sedimentation.

Photosynthetic parameters of microalgae were measured, according to Maxwell & Johnson, using a pulse-amplitude modulated (PAM) fluorometer (WaterPAM, Walz GmbH) after 10 min of dark adaptation^33^. The maximum quantum yield of PSII (F_v_/F_m_) was calculated as (F_m_ − F_0_)/F_m_, where F_0_ is the minimal fluorescence yield and F_m_ the maximal fluorescence yield under dark conditions. All measurements were performed daily. These photosynthetic parameters were obtained by irradiating the microalgae cells with pulses of saturating light.

#### Bacterial toxicity assay (*Aliivibrio fischeri*)

For the complex aquatic ecotoxicological assessment, stock suspensions (1000 mg/L) of the nanopowders and the ZnO–CuO nanocomposite with the best photocatalytic activity were prepared in distilled water and agitated at 150 rpm for 30 min at room temperature in the dark.

For the photodegradation experiments, suspensions of Rhodamine B treated with nanopowder under irradiation (TW RhB ZnO97%–CuO3% NPs) and non-irradiated controls (WRhB ZnO97%–CuO3% NPs) were examined. In addition, supernatant samples obtained after sedimentation, centrifugation (45 min, 4 °C, 4000 g; Eppendorf 5810R) and filtration (0.22 µm) were also analyzed.

The acute toxicity of the test substances to bacterial cells was evaluated using a 30-min Microtox® bioassay in accordance with ISO 11348, employing a Microtox® Model 500 Analyzer (SDI).

The freeze-dried culture of the test organism, *Aliivibrio fischeri* (DSM 7151, NRRL B-11177), was purchased from German Collection of Microorganisms and Cell Cultures (DSMZ, Leibniz Institute, Germany). *A. fischeri* is a Gram-negative, rod-shaped, facultative anaerobic, bioluminescent marine bacterium that is widely used in laboratory-based ecotoxicological testing.

Bioluminescence in *A. fischeri* is a luciferase-mediated process regulated by the *luxCDABEG* gene cluster and quorum sensing, in which FMNH₂, oxygen and an aliphatic aldehyde are converted into FMN, water and a long-chain organic acid^34,35^. Because this process directly reflects cellular metabolic activity, toxic effects result in reduced light emission; therefore, toxicity was quantified based on inhibition of bacterial bioluminescence. Prior to testing, the NaCl concentration of all test samples was adjusted to 2% (m/m). Nine-step dilution series were prepared in duplicate using a 2% (m/m) NaCl solution.

#### Crustacean toxicity assay (*Daphnia magna*)

*Daphnia magna* is a freshwater cladoceran crustacean that reproduces parthenogenetically under favorable conditions. Acute immobilization tests were conducted with neonates (< 24 h old) in accordance with OECD Test Guideline 202 to assess the effects of test materials on *D. magna* mobility and survival. *Daphnia magna* Straus stock culture was purchased from National Center for Public Health and Pharmacy (NPHCP), Department of Environmental Health Laboratories (Budapest, Hungary). Water samples were conditioned at 20–22 °C for 24 h and an artificial control medium was prepared following OECD specifications. Gravid females were isolated one day prior to testing to obtain neonates, and exposures were performed in the minimal nutrient medium specified in Annex 3 of OECD TG 202 due to potential metal chelation.

Tests were conducted at 22 ± 1 °C under a 16 h light (1000–1500 lx): 8 h dark photoperiod in a climate-controlled chamber (Memmert IPP 110). Test medium conditions were maintained within guideline limits (pH 6.5–8.5; water hardness 140–250 mg CaCO₃/L; dissolved oxygen > 3 mg/L). A decimal dilution series of the samples and a control were prepared. For each treatment, 50 mL test solution was prepared in four replicates, each containing five neonates. Immobilization and mortality were assessed after 24 and 48 h. pH and dissolved oxygen were measured at test initiation and termination, and incubator temperature was recorded daily. The percentage of immobilized or dead daphnids was determined for each concentration, and results are reported as 48 h EC₅₀ values. Test validity criteria required control mortality ≤ 10% and dissolved oxygen ≥ 3 mg/L at test termination.

#### Zebrafish embryo toxicity assay (*Danio rerio*)

The zebrafish (*Danio rerio*) is a South Asian freshwater fish widely used in developmental biology, toxicology, and drug discovery due to its rapid development, small size, and high genetic homology with humans, with approximately 70% of human protein-coding genes having zebrafish orthologues^36^.

Zebrafish embryo toxicity was assessed using the ZETA (zebrafish embryo toxicity assay). *Zebrafish* AB line was obtained from Champalimaud Foundation, Av. Brasília, 1400–038 Lisbon, Portugal. Toxicity to *Zebrafish* embryos was assessed using a slightly modified version of the Zebrafish Embryo Toxicity Assay (ZETA, Haq et al.^37^). Laboratory AB strain zebrafish were maintained at the Institute of Aquaculture and Environmental Safety of the Hungarian University of Agriculture and Life Sciences (Gödöllő, Hungary) in a Tecniplast ZebTEC recirculating system (Tecniplast Spa, Italy) in breeding groups of 30 females and 30 males. Fish were kept at 25.5 ± 0.5 °C (system water: pH 7.0 ± 0.2; conductivity 550 ± 50 µS/cm) under a 14 h light: 10 h dark photoperiod and fed twice daily with dry granulated feed (Zebrafeed 400–600 µm), supplemented twice weekly with freshly hatched *Artemia salina*.

Spawning was induced by transferring selected adults into partitioned breeding tanks on the afternoon prior to testing; partitions were removed the following morning, and fertilized eggs were collected and placed in Petri dishes containing system water.

Exposures were performed in 24-well polystyrene plates, with five embryos per well at ≤ 2 h post-fertilization (4–32 cell stage) in 2 mL aqueous test solutions. Five-step serial dilution series were applied, and four replicates were tested per concentration (n = 20). System water was used as negative control in four replicates. Embryo development was assessed at 72, 96, and 120 h post-fertilization using light microscopy. Mortality data were analyzed to calculate LC values.

The European Directive on the Protection of Animals for scientific purposes (2010/63/EU) does not apply to fish embryos before the stage of independent feeding, which begins after 120 h post-fertilization in zebrafish^38^. No independently feeding zebrafish larvae or adult animals were involved in the experiments.

### Statistical analysis

For *Scenedesmus obliquus* the data were statistically analyzed using R software. The Bartlett and Shapiro–Wilk tests were used to assess the homoscedasticity and normality of the data. When these conditions were met, ANOVA and Tukey HSD post hoc tests were used. Otherwise, non-parametric tests such as the Kruskal–Wallis test and the Wilcoxon post-hoc test were used. Results were considered significant at a *p*-value < 0.05. For *Aliivibrio fischeri* the effective concentrations causing 50% inhibition of bioluminescence (EC₅₀) after 30 min of exposure were calculated using MicrotoxOmni® software (version 1.1, AZUR Environmental Ltd.), which automatically performs data processing and endpoint calculations based on built-in standardized procedures. Graphs were created with GraphPad Prism software (GraphPad Software Inc., San Diego, USA) using the raw inhibition data measured in the Microtox® assays. In the *Daphnia magna* and ZETA assay concentration–response curve fitting, EC and LC values calculations and diagrams were generated by four-parametric logistic equation nonlinear regression model (Hill equation or variable slope sigmoidal equation with the following equation:

$$Y = Bottom + \frac{{\left( {Top - Bottom} \right)}}{{1 + 10^{{\left( {\log IC_{50} - X} \right) \cdot HillSlope}} }}$$

where *X* is the *log10*-transformed concentration, *Top* and *Bottom* are the asymptotic maximum and minimum responses, respectively, and *HillSlope* describes curve steepness) using GraphPad Prism software (GraphPad Software Inc., San Diego, USA).

## Results and discussion

### Structural and physicochemical properties

**Fig. 1:**
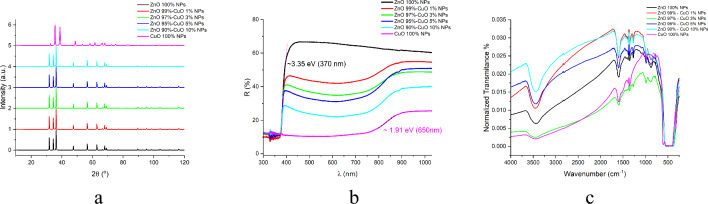
XRD patterns (**a**), Reflectance (**b**) and FTIR (**c**) spectra of metal oxide samples based on ZnO, CuO and ZnO–CuO nanocomposites.

The diffraction patterns of the investigated samples (ZnO100% NPs, ZnO99%–CuO1% NPs, ZnO97%–CuO3% NPs, ZnO95%–CuO5% NPs, ZnO90%–CuO10% NPs and CuO100% NPs) are shown in Fig. 1a. The ZnO 100% sample exhibited prominent peaks at 31.8°, 34.4°, 36.3°, 47.5°, 56.6°, 62.9°, 66.4°, 67.9°, 69.1°, 72.5° and 76.9° corresponding to (100), (002), (101), (102), (110), (103), (200), (112), (201), (004) and (202) reflections. All diffraction peaks are attributed to ZnO in the hexagonal wurtzite phase (ICDD file no. 00-036-1451). The CuO 100% NPs sample displayed prominent peaks at 32.5°, 35.4°, 35.5°, 38.7°, 38.9°, 46.2°, 48.7°, 51.3°, 53.5°, 56.7°, 58.3°, 61.5°, 65.8°, 66.2°, 66.4°, 67.9°, 68.1°, 68.9°, 71.7°, 72.4°, 72.9°, 74.9°, 75.2° and 79.7° corresponding to (110), (002), (11–1), (111), (200), (11–2), (20–2), (112), (020), (021), (202), (11–3), (022), (31–1), (310), (113), (220), (22–1), (31–2), (311), (221), (004), (22–2) and (023) reflections. All diffraction maxima are attributed to CuO in the monoclinic phase (ICDD file no. 00-048-1548). The diffraction patterns of the composite samples (ZnO99%–CuO1% NPs, ZnO97%–CuO3% NPs, ZnO95%–CuO5% NPs, ZnO90%–CuO10% NPs) exhibit peaks associated to both ZnO and CuO phases.

**Fig. 2:**
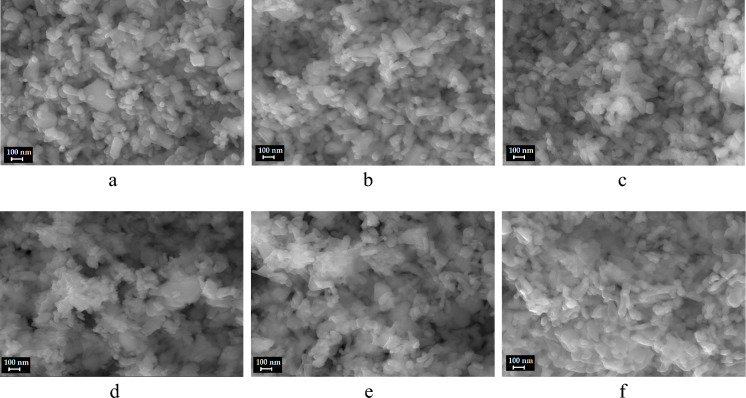
SEM images of the metal oxide samples: ZnO100% NPs (**a**); ZnO99%–CuO1% NPs (**b**); ZnO97%–CuO3% NPs (**c**); ZnO95%–CuO5% NPs (**d**); ZnO90%–CuO10% NPs (**e**); CuO100% NPs (**f**).

The SEM micrographs shown in Fig. 2 illustrate the morphological features of ZnO, CuO and ZnO–CuO composite nanoparticles highlighting the influence of CuO content on particle size, aggregation state and surface texture. All samples were examined at identical magnification (scale bar: 100 nm) allowing a direct qualitative comparison.

Pristine ZnO nanoparticles (Fig. 2a) display a granular morphology mainly composed by quasi-spherical to slightly elongated particles. The particle size may be estimated to lie predominantly in the 20–40 nm range while some elongated structures appear to extend up to 100–200 nm likely due to aggregation or oriented attachment phenomena. The incorporation of small amounts of CuO in the composite samples, namely ZnO99%–CuO1% NPs (Fig. 2b) and ZnO97%–CuO3% NPs (Fig. 2c), does not appear to induce significant changes in the overall morphology. The particle size distribution remains comparable to that observed for pristine ZnO suggesting that low CuO loading may not substantially affect the morphology of the samples. With further increasing CuO content, as observed for the ZnO95%–CuO5% NPs (Fig. 2d) and ZnO90%–CuO10% NPs (Fig. 2e) samples, the formation of larger and denser agglomerates becomes more pronounced. This evolution may indicate enhanced particle coalescence and aggregation at higher CuO concentrations. In contrast, CuO nanoparticles (Fig. 2f) exhibit a morphology clearly distinct from that of ZnO consisting of larger, poorly defined particles that are strongly agglomerated into massive clusters. The primary particle size may be estimated in the range of 40–80 nm while the aggregates can extend to several hundred nanometers. Thus, SEM analysis suggests that the controlled incorporation of CuO into the ZnO matrix may significantly influence particle morphology and aggregation behavior. Lower CuO contents (1–3%) appear to promote better-dispersed nanostructures whereas higher CuO concentrations favor the formation of compact agglomerates. These morphological trends may help explain the existence of an optimal CuO loading in the ZnO–CuO composite samples.

Figure S1 presents the EDX elemental mapping and corresponding spectra for the ZnO–CuO composites. The elemental maps confirm the presence and spatial distribution of Zn, Cu and O across the analyzed areas for all samples. The EDX mapping analysis proves the successful incorporation of CuO into the ZnO matrix and reveals a clear evolution of elemental distribution with increasing of CuO content. Low and intermediate CuO loadings (1–3%) favor a more homogeneous dispersion of CuO promoting extensive ZnO–CuO interfacial contact. In contrast, higher CuO contents (≥ 5%) tend to induce compositional inhomogeneity and CuO aggregation, which may adversely affect photocatalytic performance by reducing effective interfacial area and active surface accessibility. These observations are in good agreement with the SEM results and support the existence of an optimal CuO loading in the ZnO–CuO composite system.

Based on the reflectance data (Fig. 1b), the estimated band gap energies were approximately at ~ 3.35 eV for ZnO and ~ 1.91 eV for CuO, both values being in accordance to those reported in literature^39–41^. The reflectance spectra of the composite samples (ZnO99%–CuO1% NPs, ZnO97%–CuO3% NPs, ZnO95%–CuO5% NPs, ZnO90%–CuO10% NPs) display two distinct slopes corresponding to ZnO and CuO. As expected, ZnO primarily absorbs in the ultraviolet region (~ 370 nm) while CuO absorbs in the visible region (~ 650 nm). Therefore, solar light (visible range) can be effectively used for irradiation in the photocatalytic experiments.

The FTIR spectra (Fig. 1c) of ZnO and CuO samples exhibited a band at ~ 542 cm⁻^1^ attributed to the characteristic stretching vibration of the Zn–O bond^42,43^ and a band at ~ 598 cm⁻^1^ associated to the stretching vibration of the Cu–O bond^10,44^, respectively. The ZnO–CuO composites display an intermediate spectral profile between the two individual metal oxide powders, the intensity and broadening of the metal–oxygen bands varying as the CuO content increases, confirming the contribution of each oxide phase within the composite. All FTIR spectra display a FTIR band centered around ~ 3447 cm⁻^1^ (stretching mode) assigned to O–H stretching vibrations originating from adsorbed water molecules or surface hydroxyl groups, which are typical for commercial nanopowders stored at room temperature^40,45^. The band around 1600 cm⁻^1^ corresponds most likely to O–H bending mode of adsorbed water molecules. The peaks in the region 1500–1300 cm⁻^1^ are often resulted from the adsorption of atmospheric CO_2_ on the surface of metal oxides^46^.

DLS measurements showed ZnO particles with ~ 73 nm average size. The results indicate that the ZnO100% NPs sample exhibits a monomodal distribution with particles predominantly below 100 nm and a mean hydrodynamic diameter closely matching the manufacturer’s specifications (Fig. S2). This narrow size distribution suggests a high degree of dispersion and minimal agglomeration. Conversely, the CuO100% NPs sample presents a bimodal distribution consisting of two distinct nanoparticle populations. The first population (55.3%) has an average particle size of 9.4 nm, consistent with the nominal specification of < 50 nm. However, the second population (44.7%) shows a significantly larger particle size around 274.9 nm, which is indicative of nanoparticle agglomeration phenomena that can be attributed to the tendency of nanoparticles to agglomerate under certain storage or dispersion conditions. This behavior is commonly observed in metal oxide systems with high surface energy and insufficient electrostatic stabilization, leading to cluster formation in aqueous or low-polarity suspensions^47^. Such agglomeration can have direct implications for the optical, catalytic and surface properties of the CuO-based materials. Larger agglomerates tend to reduce the effective surface area and may limit photogenerated charge separation efficiency, thus negatively impacting photocatalytic performance. On the other hand, some degree of agglomeration can enhance light scattering and absorption pathlength, which might be beneficial in specific optical applications^48^. Moreover, the differences in dispersion behavior between ZnO and CuO particles could affect the homogeneity and interfacial interactions in the composite materials. These interactions are critical for synergistic effects in heterojunction systems, where electron transfer dynamics at the oxide interface play an important role in determining the efficiency of charge separation and recombination processes^49^. As observed, the composite ZnO99%–CuO1% NPs exhibited a monomodal distribution of particle size and a mean hydrodynamic diameter of 91.46 nm while the other composites (ZnO97%–CuO3% NPs, ZnO95%–CuO5% NPs and ZnO90%–CuO10% NPs) showed a bimodal size distribution and a mean hydrodynamic diameter with values < 74 nm (Fig. S2). The DLS results are consistent with the SEM observations, in that the nanoscale primary particles coexist with larger secondary agglomerates in dispersion, with the bimodal size distributions and increased hydrodynamic diameters reflecting aggregation phenomena that become more pronounced as CuO content increases.

**Fig. 3:**
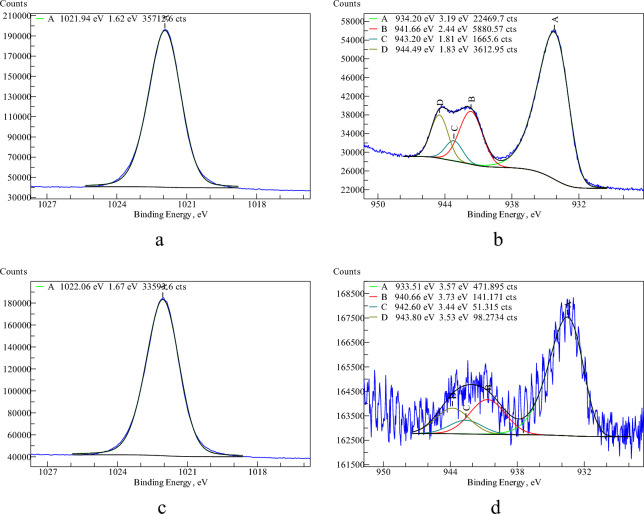
XPS spectra of Zn2p3/2 for ZnO NPs (**a**), ZnO97%–CuO3% NPs (**c**) samples and of Cu2p3/2 for CuO NPs (**b**) and ZnO97%–CuO3% NPs (**d**) samples.

XPS measurements were used to determine the chemical structure of the surface of the samples. Survey spectra (Fig. S3), core level spectra of C1s, O1s, Zn2p_3/2_, Cu2p_3/2_ and where possible the main Auger lines Zn LMM and Cu LMM were recorded. The calculated surface composition from XPS analysis shows a ratio of 97.6:2.4 for Zn:Cu in ZnO97%–CuO3% NPs sample (Table S1). The peak associated with C–C bonds in the C1s spectra was used as a charge reference for all other XPS spectra and set at binding energy of 284.8 eV (Fig. S4). The O1s spectra also show similarities across all three samples (Fig. S5). The presence of the metallic oxides is clearly visible in the O1s spectra of the ZnO NPs, CuO NPs and ZnO97%–CuO3% NPs samples (Fig. S5). The comparison between ZnO97%–CuO3% NPs and the simple oxide samples shows some notable differences in the binding energies of Zn2p_3/2_ and Cu2p_3/2_ (Fig. 3). The spectra of Zn2p_3/2_ are fitted with only one symmetric peak at binding energies usually found in the literature for ZnO (Fig. 3a, c)^50^. In the case of Cu2p_3/2_ spectra presence of the shake-up satellites (Fig. 3b, d) the peaks B, C and D, clearly confirm the presence of Cu^2+^ oxidation state. The binding energy of main peaks A (Fig. 3b, d) is attributed to CuO compound^51,52^. The calculated modified Auger parameters for Zn and Cu have the value of 2010.36 eV for Zn and 1852.03 eV for Cu, respectively. These values also certify the ZnO and the CuO compounds (Fig. S6)^51^. The modified Auger parameter was calculated as a sum between binding energy of 2p_3/2_ line and the kinetic energy of the maximum of LMM Auger spectra (Fig. S6). The Zn2p_3/2_ spectrum shows a very slight increase of approx. 0.1 eV in the binding energy in the composite sample compared to the pristine ZnO NPs sample. The difference is even greater in the case of Cu2p_3/2_ spectrum of composite sample compared with pristine CuO NPs sample. In the case of ZnO97%–CuO3% NPs sample, the binding energy decreases by almost 0.7 eV. These shifts of binding energies of Zn2p_3/2_ and Cu2p_3/2_ show a transfer of electronic charge from ZnO (n-type) to CuO (p-type) which confirm the formation of an intimate junction between them. In accordance with the XPS results, the formation of the p–n heterojunction between the two metal oxides was evaluated by PL measurements. Thus, the PL spectrum of the ZnO sample (Fig. S7) shows a weak UV emission (~ 380 nm) and a dominant broad visible band associated with defect-related states (~ 510–530 nm)^53^. The ZnO97%–CuO3% NPs nanocomposite exhibits a significantly reduced PL intensity suggesting a suppressed electron–hole recombination and a more efficient charge separation due to the p–n heterojunction^54,55^.

**Fig. 4:**
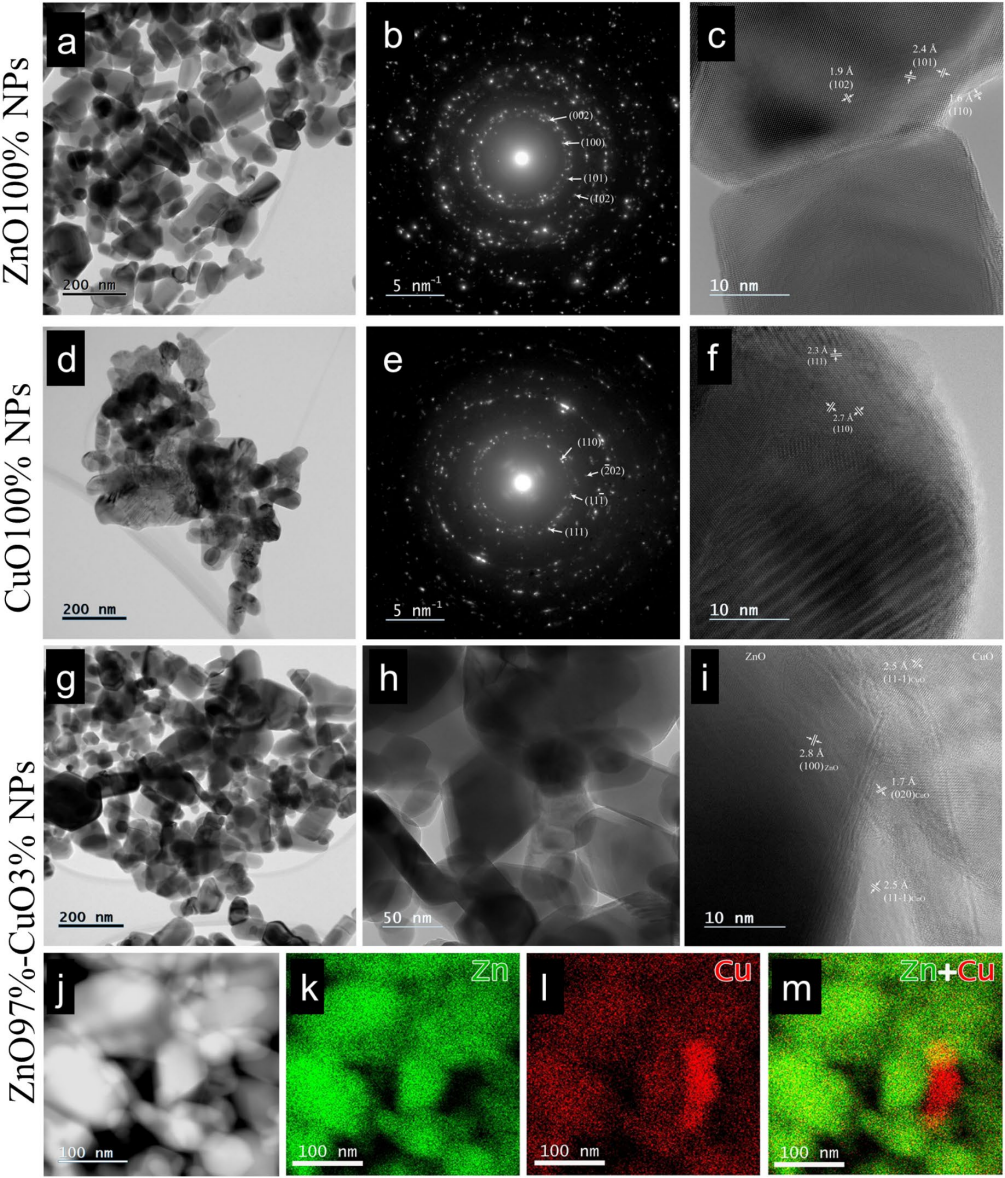
CTEM images for ZnO100% NPs (**a**), CuO100% NPs (**d**) and ZnO97%–CuO3% NPs (**g**) and (**h**); SAED patterns for ZnO100% NPs (**b**) and CuO100% NPs (**e**); HRTEM images for ZnO100% NPs (**c**), CuO100% NPs (**f**) and ZnO97%–CuO3% NPs (**i**) and HAADF—STEM image (**j**), elemental maps of Zn (**k**) and Cu (**l**) and the overlay of two maps (**m**) for ZnO97%–CuO3% NPs sample.

Conventional TEM (CTEM) images for both ZnO100% NPs and CuO100% NPs (Fig. 4a, d) show nanoparticles with diverse morphologies. For ZnO100% NPs, the longest measured particle size varies between 26 and 235 nm, while for CuO 100% NPs, it ranges from 18 to 340 nm. Selected Area Electron Diffraction (SAED) patterns with diffraction spots corresponding to (100), (002), (101), (102) planes (Fig. 4b) characteristic to hexagonal ZnO and to (110), (11–1), (111), (− 202) planes (Fig. 4e) characteristic to monoclinic CuO. High Resolution TEM (HRTEM) images (Fig. 4c, f) demonstrate that nanoparticles for both samples are well crystallized. In these images, the lattice fringes of 2.4 Å, 1.9 Å and 1.6 Å corresponding to (101), (102), (110) planes of hexagonal ZnO and of 2.7 Å, 2.3 Å corresponding to (110), (111) planes of monoclinic CuO are clearly visible. CTEM images of ZnO97%–CuO3% NPs (Fig. 4g, h) show no variations of the morphology of nanoparticles compared with those from Fig. 4a, d. HRTEM image (Fig. 4i) illustrate an area of a heterojunction previously determined using EDX in STEM mode. In this image, on the right side lattice fringes 2.5 Å, 1.7 Å corresponding to (11–1), (020) planes of monoclinic CuO can be observed, while on the left side lattice fringes of 2.8 Å corresponding to (100) plane of hexagonal ZnO are visible. High Angle Annular Dark Field (HAADF) image (Fig. 4j) shows the area used for obtaining the elemental maps of Zn (Fig. 4k) and Cu (Fig. 4l). The overlap of the two maps (Fig. 4m) demonstrates the existence of the heterojunction.

### Photocatalytic activity

**Fig. 5:**
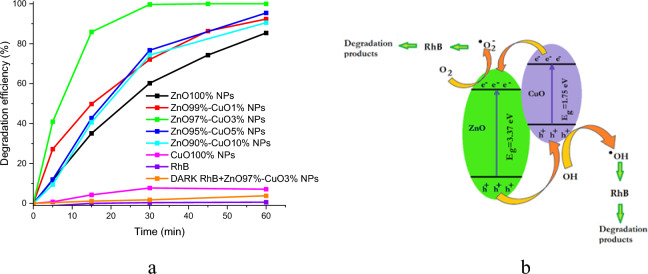
Photodegradation of pollutants in synthetic wastewater containing RhB dye (10⁻^5^ M) by metal oxides based on ZnO, CuO and ZnO–CuO nanocomposites (**a**); Schematic representation of the RhB degradation mechanism by ZnO–CuO nanocomposites (**b**).

Further, the photocatalytic activity of the pristine ZnO, CuO and ZnO–CuO composite powders was evaluated using Rhodamine B (RhB) as a model pollutant under visible light irradiation. As shown in Fig. 5, no dye degradation occurs under visible light in the absence of the photocatalyst, while all ZnO–CuO composite samples exhibited enhanced photocatalytic performance compared to the monocomponent ZnO and CuO powders, highlighting the beneficial role of ZnO–CuO heterojunction formation. The negligible dye removal observed under dark conditions, compared to the pronounced degradation under irradiation, may indicate that adsorption plays a minor role, with photocatalysis being the dominant mechanism.

As shown in Fig. 5a, the ZnO97%–CuO3% NPs nanocomposite exhibits the highest photocatalytic degradation efficiency, reaching the fastest and most pronounced RhB removal among all investigated samples, indicating the presence of an optimal CuO content. At low CuO loading (1 wt%), the photocatalytic performance is only slightly improved compared to pristine ZnO, suggesting that the amount of CuO is insufficient to establish an effective network of ZnO–CuO interfaces, resulting in limited p–n heterojunction formation and dominant charge carrier recombination. By increasing the CuO content to 3 wt% significantly enhances the density of ZnO–CuO interfacial contacts promoting efficient charge separation across the p–n heterojunction. In this regime, CuO can act as an electron acceptor suppressing electron–hole recombination and prolonging charge carrier lifetimes, which favors the generation of reactive oxygen species responsible for RhB degradation. Importantly, at this intermediate CuO loading, the ZnO surface remains largely accessible to light irradiation and dye adsorption. At higher CuO contents (≥ 5 wt%), the photocatalytic efficiency no longer improves and may even decrease. This behavior can be attributed to light-shielding effects, partial blocking of ZnO active sites and increased CuO–CuO aggregation, as evidenced by SEM and EDX analyses (Figs. 2d–e and S1c–d), which may introduce additional recombination centers and reduce the effective interfacial area. As a result, the ZnO–CuO composite containing 3 wt.% CuO represents an optimal compromise between heterojunction-driven charge separation and minimized recombination and surface-blocking effects, in agreement with previously reported behavior for heterostructured metal oxide photocatalysts^56^ (Table 1).

**Table 1:** Comparison of photocatalytic efficiency of various ZnO–CuO nanocomposites toward RhB degradation.

Photocatalyst	Light source Power	Dye (RhB) concentration	Photocatalyst dose	Degradation (%)	Time (min)	Refs.
ZnO–CuO (10%–CuO)	Solar simulator with an intensity of 300 W	2 × 10⁻^5^ M	1 mg/1 mL	93	240	^13^
ZnO–CuO (50%–CuO)	LED lamp Philips energy saver 40W	7.3 × 10⁻^5^ M	2 mg/1 mL	98.34	66	^57^
ZnO–CuO (2%–CuO)	Xe lamp 250W	2.1 × 10⁻^5^ M	1 mg/1 mL	97.2	240	^56^
ZnO–CuO (3%–CuO)	Solar simulator–xenon lamp 300 W	10⁻^5^ M	1 mg/1 mL	99.65	30	This work

The photocatalytic trends discussed above are consistent with the morphological and compositional features revealed by SEM and EDX analyses, which indicate an optimal Cu dispersion and extensive ZnO–CuO interfacial contact at 3 wt.% CuO, in contrast to the increased aggregation and interfacial loss observed at higher CuO loadings. The enhanced photocatalytic activity of ZnO–CuO heterostructures has been widely attributed to improved interfacial charge separation and extended visible light absorption. Recent studies on p–n CuO–ZnO heterojunctions demonstrated that the formation of intimate semiconductor contacts promotes efficient separation of photo-generated electron–hole pairs, thereby increasing degradation rates of organic dyes compared to individual oxide^58^. Thus, in our work, XPS results (Fig. 3), PL data (Fig. S7) and TEM images (Fig. 4) evidence the formation of p–n heterojunction interfaces, which enhance the charge separation resulting in higher photocatalytic degradation efficiency in the case of the composite sample. Moreover, reviews on visible-light-active ZnO-based photocatalysts emphasize that heterostructure design is a key strategy for reducing recombination and improving performance under solar-spectrum illumination^59^. Similar findings in S-scheme CuO–ZnO systems further support the role of CuO as an electron acceptor to suppress recombination^60^. Thus, under solar light irradiation, electrons (e⁻) and holes (h⁺) are generated in both CuO and ZnO. Due to the lower conduction and valence band positions of ZnO compared to CuO, electrons can transfer from CuO to ZnO while holes migrate from ZnO to CuO (Fig. 5b). Oxygen molecules in the dye solution react with electrons (e⁻) to generate superoxide radicals (∙O₂⁻) and the holes (h⁺) react with water molecules (H₂O) to form hydroxyl radicals (∙OH). Both superoxide (∙O₂⁻) and hydroxyl (∙OH) radicals are highly reactive species capable of degrading organic pollutants such as RhB into less harmful compounds. These radicals are able to break down complex organic molecules into smaller and non-toxic substances. In conclusion, the combined mechanism of photo-induced charge generation, interfacial charge transfer between CuO and ZnO, and reactive oxygen species (ROS) formation makes this composite a highly efficient photocatalyst for the degradation of organic pollutants under solar light exposure. After 1 h of irradiation, for the treatment of wastewater containing RhB dye (Fig. 5a), the best result was obtained for the ZnO97%–CuO3% composite.

### Nanoparticle dissolution behavior

**Fig. 6:**
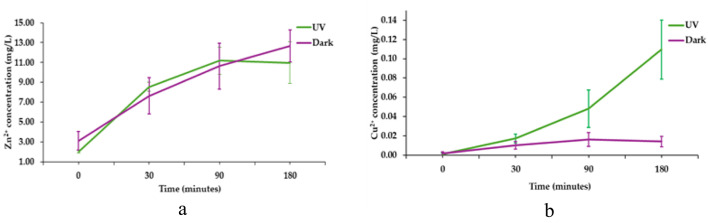
Zn^2+^ release from ZnO97% NPs (**a**) and Cu^2+^ release from CuO3% NPs (**b**) expressed as free ions concentrations measured during the dissolution kinetics of ZnO97%–CuO3% NPs (mean ± S.D., n = 3) in deionized water.

However, photocatalytic efficiency alone does not ensure environmental compatibility. Therefore, the intrinsic dissolution behavior of the ZnO–CuO composite with the best photocatalytic activity (ZnO97%–CuO3%) was investigated through dissolution kinetics experiments performed in deionized water under dark conditions and UV irradiation (365 nm). Deionized water was chosen as a chemically simple matrix to isolate the effects of irradiation on nanoparticle dissolution without interference from complexing agents or inorganic ligands. Nanoparticle suspensions were sampled at four time points between 0 and 180 min. Samples were centrifuged and the supernatants were subsequently ultrafiltered to isolate the dissolved metal fraction. This procedure allowed quantification of free Zn^2+^ and Cu^2^⁺ ions released from the nanoparticles. For the ZnO NPs fraction (ZnO97%), measurable concentrations of dissolved zinc (between 2 and 3 mg/L) were detected at the initial sampling time suggesting either rapid early dissolution or the presence of dissolved zinc originating from the nanoparticle stock suspension (Fig. 6a). During the 180-min exposure period, Zn^2+^ concentrations increased similarly under both dark and UV conditions reaching 10.98 ± 2.09 mg/L under UV irradiation and 12.68 ± 1.61 mg/L in the dark. These results indicate that, under the tested conditions, UV irradiation did not significantly enhance ZnO dissolution in deionized water. In contrast, CuO dissolution remained negligible under dark conditions and at the initial time point (Fig. 6b). A measurable release of Cu^2+^ was observed only under UV irradiation reaching 0.11 ± 0.03 mg/L after 180 min indicating a limited but detectable influence of irradiation on CuO dissolution. The results confirm that the ZnO–CuO nanocomposites predominantly release zinc ions in deionized water, while copper ion release remains low. The measured dissolved zinc concentrations are comparable to literature values with a dissolution of 7.18–7.40 mg/L obtained in nanopure water using a similar protocol^31^. However, this ion release represents a non-negligible fraction of the total zinc content (> 10%) and falls within concentration ranges known to induce adverse effects in aquatic organisms^61,62^.

To assess the dissolution behavior of the ZnO–CuO composite with the best photocatalytic activity (ZnO97%–CuO3%) under more realistic conditions, dissolution experiments were extended from deionized water to Bold’s Basal Medium (BBM) under dark conditions. BBM was selected as a representative complex matrix containing salts, nutrients, and organic compounds commonly found in aqueous environments. This approach enabled a comparison between dissolution rates in a chemically simple medium (deionized water) and a complex nutrient-rich matrix (BBM).

**Fig. 7:**
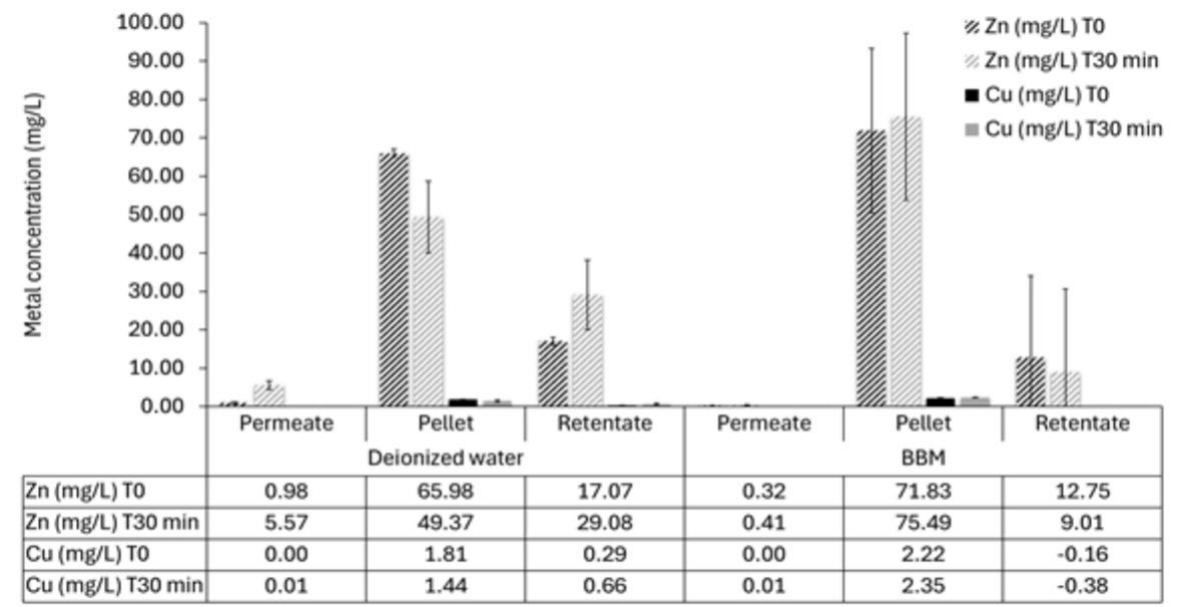
Mass balance of zinc and copper distribution between pellet, permeate (< 3 kDa) and calculated losses during ZnO97%–CuO3% NPs dissolution (mean ± S.D., n = 3) in Bold’s Basal Medium (BBM). Permeate, pellet and estimated metal remaining in the retentate are shown.

A mass balance approach was employed to quantify zinc and copper distribution between dissolved and particulate fractions after 30 min of incubation. Samples were centrifuged to separate nanoparticles and precipitates from the soluble fraction, and ultrafiltration (3 kDa) was used to isolate free dissolved ions. Metal concentrations were quantified by ICP-AES. Zinc dissolution in BBM (0.41 ± 0.03 mg/L) was substantially lower than in deionized water (5.57 ± 1.19 mg/L) after 30 min (Fig. 7) demonstrating that medium composition strongly influences nanoparticles dissolution quantification. This reduced apparent dissolution indicates that the quantification of dissolved ions in BBM is constrained by medium-dependent processes.

Although ethylenediaminetetraacetic acid (EDTA) and other organic ligands are generally reported to enhance ZnO nanoparticle dissolution^17,19^ the present results suggest that, in this complex medium, dissolved zinc cannot be interpreted solely based on chelation effects in this complex medium. Instead, multiple concurrent processes likely limit the direct observation of free dissolved ions highlighting a methodological limitation inherent to complex culture media. Consistent with previous results (Fig. 6b), no copper was detected in the permeates of either matrix at T0 or T30 min, confirming the limited dissolution of CuO under these conditions. The mass balance analysis revealed that the majority of zinc in BBM remained associated with the particulate fraction, either as undissolved nanoparticles or as newly formed precipitates, in contrast to the substantial free ion release observed in deionized water. These findings highlight the importance of evaluating nanoparticle dissolution in chemically diverse matrices because medium composition critically governs metal ion speciation and availability. Understanding these dissolution patterns and the chemical forms of released metals is useful for assessing the environmental behavior of metal oxide nanoparticles in complex aqueous systems.

### Ecotoxicological assessment

#### Plant physiological responses (*Ocimum basilicum*)

In the following, we examined the relevant aspects of plant physiology because these are essential for understanding how plants respond when exposed to different water types.

The basil plants were fed with aqueous suspensions containing powders (1 g/L) of metal oxides (ZnO100% NPs and CuO100% NPs) and their composite ZnO97%–CuO3% NPs. The control plants were fed only with water.

**Fig. 8:**
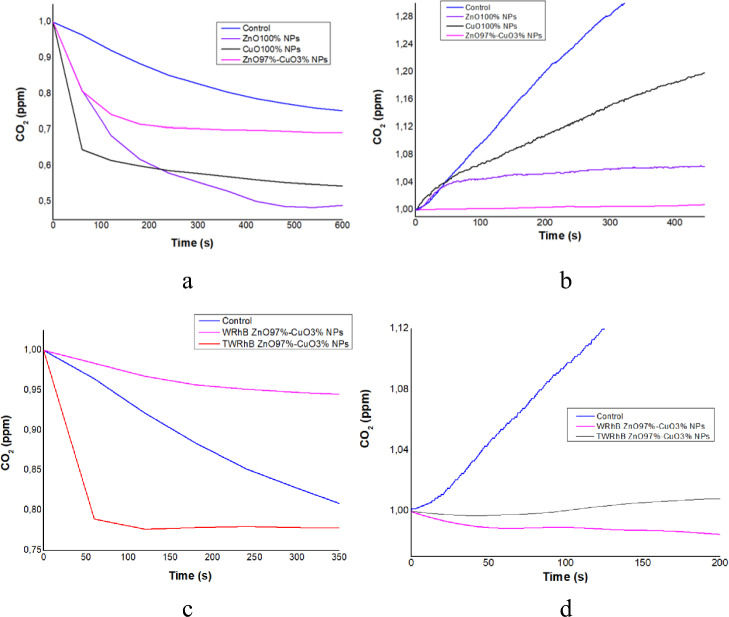
Photosynthesis (**a**) and respiration (**b**) in untreated plants (control) and in plants exposed to aqueous suspensions (1 mg/mL) of ZnO100% NPs, CuO100% NPs, and ZnO97%–CuO3% NPs. Photosynthesis (**c**) and respiration (**d**) in untreated plants (control) and after treatment with WRhB ZnO97%–CuO3% NPs and TW RhB ZnO97%–CuO3% NPs. Data were normalized to facilitate comparison.

The collected CO₂ data allowed for the assessment of photosynthetic CO₂ uptake and respiratory CO₂ release in *Ocimum basilicum L.* plants under different treatments. Variations in CO₂ concentration over time reflected the net balance between photosynthesis (CO₂ consumption) and respiration (CO₂ release). During the photosynthesis process, the decrease in CO₂ concentration over time was monitored (Fig. 8a). The assessment of photosynthetic activity in plants treated with aqueous suspensions of metal oxides and their composites, specifically ZnO97%–CuO3% NPs, revealed over the significant time intervals analyzed a decrease in CO₂ concentration (ppm). This confirms the plants’ ability to perform photosynthesis with minor variations depending on the sample type. It is noteworthy that in the case of the sample treated with CuO100% NPs suspension, there was a sharp initial decrease in CO₂ concentration during the early phase of the monitored sequence (Fig. 8a) followed by a linear downward trend. This pattern was observed across all tested samples including the control. This behavior may be explained by the stimulatory effect of CuO nanoparticle suspensions on plant metabolism and consequently on the photosynthetic process itself.

The plant respiration was evaluated by monitoring the CO₂ concentration (ex-pressed in ppm) over a period of 120–180 min under low light conditions (darkness) and at room temperature. During the plant respiration process, the increase in CO₂ concentration over time was monitored and recorded (Fig. 8b). The gradual increase in CO_2_ concentration over time was consistent across all tested samples, confirming the metabolic viability and active respiration of the treated plants (Fig. 8b). The progressive increase in CO₂ concentration corresponds to mitochondrial respiration activity during which carbohydrates are oxidized to release energy (adenosine triphosphate (ATP)) and CO_2_ as a byproduct. Under dark conditions, photosynthesis ceases making respiration the dominant gas-exchange process^63^. All tested plants showed CO₂ accumulation over time confirming their metabolic viability. In plants treated with CuO100% NPs, the initial rate of CO₂ increase was steeper suggesting an enhanced respiratory activity, possibly triggered by oxidative stress or metabolic stimulation from CuO nanoparticle exposure^63,64^. In the evaluation of the plant respiration process, an increase in CO₂ concentration (ppm) was observed across pristine samples. However, a slightly different trend was noted in the case of plants treated with ZnO97%–CuO3% NPs suspension. This behavior may be attributed to the presence of 3% CuO nanoparticles, which appear to enhance resistance to specific environmental conditions, particularly low-light (dark) environments. Interestingly, the initial phase of monitoring exhibited a temporary decrease in CO₂ concentration, a phenomenon typically associated with photosynthetic activity.

The basil plants were fed with synthetic wastewater WRhB ZnO97%–CuO3% NPs and TW RhB ZnO97%–CuO3% NPs. The control plants were fed only with water. The monitoring period ranged between 120 and 180 min. A representative time interval was selected in order to highlight the photosynthesis and respiration processes in the plants. Thus, the analysis of synthetic wastewater treated with metal oxide powders showed that the presence of RhB in the water used for plant irrigation led to a deterioration of photosynthesis and respiration processes, ultimately causing the premature death of the plants (Fig. S8).

The evaluation of the photosynthesis in untreated plants (Fig. 8c) as well as in plants fed with synthetic wastewater containing RhB and ZnO97%–CuO3% NPs (unirradiated) and irradiated RhB solution and ZnO97%–CuO3% NPs reveals a decrease in CO₂ concentration (ppm) over the analyzed time intervals, indicating the ability of the plants to perform photosynthesis. However, the photosynthetic behavior varies depending on the type of solution used (distilled water, RhB solution before photodegradation or RhB solution post-photodegradation). Specifically, in the case of irrigation with RhB solution containing ZnO97%–CuO3% NPs (before photodegradation), the photosynthetic process is slowed down indicating a phytotoxic effect making it difficult for the plant to sustain normal photosynthetic activity (Fig. 8c). In contrast, when the plant is fed with RhB solution that was photocatalytically degraded using ZnO97%–CuO3% NPs, the photosynthetic behavior is closely aligned to that of the control plant irrigated with distilled water (Fig. 8c). In the case of plant respiration, a similar trend is observed. In these experiments, the increase in CO₂ concentration (ppm) was monitored following the expected pattern under dark conditions (Fig. 8d). As observed, the plants fed with untreated RhB solution exhibited abnormal respiration process while those treated with TW RhB ZnO97%–CuO3% NPs showed normal respiratory behavior. Thus, the analysis of synthetic wastewater treated with metal oxide powders showed that the presence of RhB in the water used for plant irrigation led to a deterioration of photosynthesis and respiration processes, ultimately causing the premature death of the plants. In these experiments, specific sequences were captured (Fig. 8) in which the photosynthesis and respiration processes were recorded because distinct physiological responses were observed depending on the treatment type. In conclusion, the plant has a biochemical behavior and a particular response for each type of treatment. Thus, monitoring both photosynthesis and plant respiration processes involves capturing them at different times and also at different CO_2_ concentrations. It has been observed that both metal oxides NPs, especially CuO NPs, have beneficial effects on photosynthesis and respiration processes. Mahmood et al.^65^ highlighted the copper is an essential element for chlorophyll biosynthesis and photosystem stability. Moreover, zinc and copper are essential nutrients for crops playing important roles in photosynthesis and respiration processes^66,67^. Also, Mahawar et al. revealed that ZnO NPs and CuO NPs have beneficial effects in promoting growth and reducing the adverse effects of NaCl stress in radish plants^68^.

#### Microalgae response (*Scenedesmus obliquus*)

To assess whether the presence of ZnO–CuO composite with the best photocatalytic activity (ZnO97%–CuO3% NPs) could affect microalgal growth directly or indirectly through ion release, growth assays were designed to distinguish between particulate and dissolved metal effects. *Scenedesmus obliquus* was therefore exposed in BBM to the selected ZnO97%–CuO3% NPs at 100 mg/L, a concentration representative of the photocatalytic assays. In parallel, microalgae were exposed to dissolved zinc at a concentration corresponding to the maximum ZnO dissolution measured under these conditions (12.5 mg Zn^2+^/L). Copper ions were not specifically tested, as CuO dissolution was negligible and BBM already contains Cu^2+^ at concentrations exceeding those released from the nanoparticles. This experimental design allows the potential contribution of nanoparticle-associated effects and dissolved zinc to microalgal responses to be evaluated under comparable and environmentally relevant culture conditions.

**Fig. 9:**
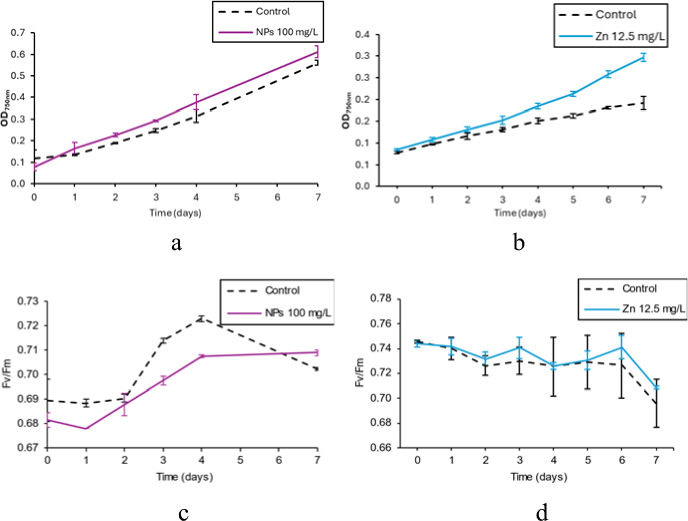
*Scenedesmus obliquus* growth curves in the presence of: 100 mg/L ZnO97%–CuO3% NPs (mean ± S.D., n = 2) (**a**) and 12.5 mg/L Zn^2+^ (mean ± S.D., n = 3) (**b**). Photosynthetic efficiency (Fv/Fm) of *Scenedesmus obliquus* exposed to: 100 mg/L ZnO97%–CuO3% NPs (mean ± S.D., n = 2) (**c**) and 12.5 mg/L Zn^2+^ (mean ± S.D., n = 3) (**d**).

As shown in Fig. 9a, similar absorbance profiles were observed over the 7-day cultivation period for control cultures and for cultures exposed to the nanoparticle suspension indicating that microalgal growth was not impaired by the presence of nanoparticles at the tested concentration. In contrast, cultures exposed to dissolved Zn^2+^ exhibited a significantly higher absorbance at the beginning of the experiment (0.30 ± 0.01) compared to the control (0.19 ± 0.02; *p* < 0.05) suggesting that zinc ions did not inhibit growth and may even have exerted a stimulatory effect under these conditions (Fig. 9b). Importantly, no growth inhibition was observed after 7 days of exposure, either in the presence of nanoparticles or dissolved zinc.

These observations were further supported by measurements of the maximum quantum yield of photosystem II (Fv/Fm, where Fv is variable fluorescence and Fm is maximum fluorescence), which remained comparable to control values after 7 days. This indicates that neither the nanoparticles nor the released ions induced a detectable decrease in photosynthetic efficiency (Fig. 9c, d). These results suggest that, in BBM, *S. obliquus* tolerates both the nanoparticle suspension and the associated dissolved zinc concentrations without measurable adverse effects on growth or photosynthetic efficiency.

This observation appears to contrast with some reports in the literature. For instance, Zhou et al. reported a significant toxicity of Zn^2^⁺ to *Scenedesmus obliquus* at concentrations as low as 5 mg/L in a modified blue green medium (BG11) suggesting that the BBM culture medium may modulate the physiological response of microalgae to dissolved metal ions by mitigating metal-induced stress^69^. In contrast, the results reported by Aravantinou et al*.* partly support our observations as they showed that *Scenedesmus rubescens* was more strongly affected by ZnO nanoparticle toxicity in BG11 than in BBM despite exhibiting higher growth rates in BG11 under control (non-exposed) conditions^70^. This study highlights that the culture medium not only influences nanoparticle dissolution and the apparent availability of dissolved ions but also plays a key role in determining microalgal sensitivity to metal-related stress. In this context, BBM appears to provide a more protective chemical environment, reducing the adverse effects associated with both ZnO nanoparticles and dissolved Zn^2+^ ions.

Therefore, the present results indicate that BBM strongly modulates the biological response of *S. obliquus* to both nanoparticles and dissolved zinc. They underline the importance of considering matrix effects when assessing nanoparticle toxicity and highlight the need for further investigations focusing on realistic aqueous media, nanoparticle dissolution behavior, metal speciation and species-specific sensitivity under environmentally relevant conditions.

#### Aquatic toxicity assays

In *Aliivibrio fischeri*, nano-ZnO powder caused 50% inhibition of bioluminescence at 170 mg/L whereas nano-CuO powder showed no detectable toxicity within the tested concentration range. *Daphnia magna* was more sensitive to metal oxides with EC₅₀ values of 16.2 mg/L for ZnO and 28.7 mg/L for CuO.

**Fig. 10:**
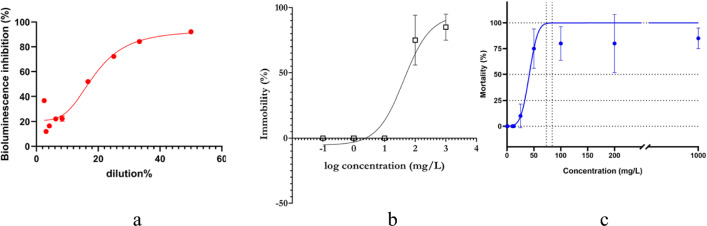
Concentration–response curves for ZnO97%–CuO3% composite in the aquatic ecotoxicological assays. *Aliivibrio fischeri* acute bioluminescence inhibition (%) (**a**); *Daphnia magna* acute immobilization (%) (**b**); *Danio rerio* embryo mortality (%) (**c**).

For the ZnO–CuO composite (ZnO97%–CuO3% NPs), *A. fischeri* was the least sensitive organism (EC₅₀ = 140 mg/L) while *D. magna* and *Danio rerio* embryos exhibited comparable sensitivities with EC₅₀ values of 42.6 and 34.1 mg/L, respectively (Fig. 10).

Residues obtained from RhB photodegradation using the ZnO97%–CuO3% NPs composite (TW RhB ZnO97%–CuO3% NPs) as well as the non-irradiated control (WRhB ZnO97%–CuO3% NPs) were toxic to all aquatic test organisms. Effective concentrations causing 50% effects, expressed as percentages of the original (100%) sample, were as follows: for *Aliivibrio fischeri*, 34.6% of the control sample and 21.5% of the irradiated degradation residue caused 50% inhibition of bioluminescence; for *Daphnia magna*, 37.1% of the control and 58.6% of the treated sample resulted in 50% immobilization. In zebrafish (*Danio rerio*) embryos, both samples caused 100% mortality at the lowest tested dilution (6.25%).

**Fig. 11:**
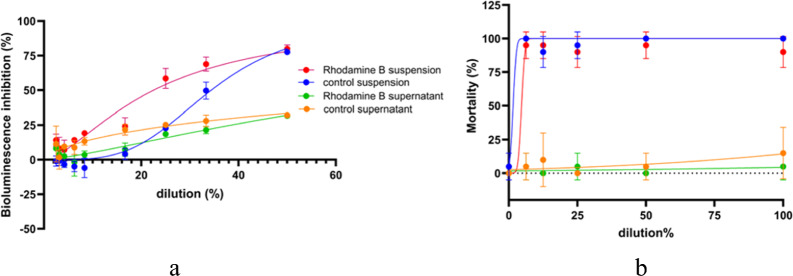
Comparison in toxicity between the suspension and supernatant of RhB degradation sample (TW RhB ZnO97%–CuO3% NPs) and untreated control (WRhB ZnO97%–CuO3% NPs). *Aliivibrio fischeri* acute bioluminescence inhibition (%) (**a**); *Danio rerio* embryo mortality (%) (**b**).

Following sedimentation and filtration of nanopowder-containing samples, the toxicity of the supernatants was also evaluated. A pronounced reduction in toxicity was observed in the *A. fischeri* and zebrafish assays compared to the corresponding suspensions. In the *A. fischeri* test, 131.3% and 127.5% of the control and treated supernatants, respectively, were required to cause 50% bioluminescence inhibition, while the supernatants were essentially non-toxic in the zebrafish embryo test (Fig. 11). In contrast, no substantial reduction in toxicity was observed for *D. magna*, highlighting its elevated sensitivity to dissolved ionic species potentially remaining in the supernatant.

The acute toxic effects of CuO and ZnO nanoparticles in *Daphnia magna* have been well documented^71,72^. According to previous studies, *Daphnia magna* exhibits high sensitivity to CuO nanoparticles, with 48-h EC_50_ values ranging from 3.2 to 22 mg/L. For ZnO nanoparticles, EC_50_ and LC_50_ values range from 0.99 to 25 mg/L. These toxicity thresholds depend on factors such as nanoparticle size (< 50 nm), synthesis method, pH, water hardness, organic matter concentration and nanoparticle aging. Nanoparticles generally demonstrate greater toxicity than bulk metal forms but are less toxic than dissolved metal ions. Dissolved metal ions are typically considered the primary toxicants, with greater potency than nanoparticles. In particular, chronic toxicity studies on *Daphnia magna* have demonstrated that ZnO nanoparticle toxicity is largely driven by dissolved Zn^2^⁺ with significant biological effects occurring at concentrations as low as 1–5 mg/L^73^. These findings highlight the necessity of quantifying nanoparticle dissolution as a prerequisite for interpreting toxicity outcomes and for distinguishing between particle-specific effects and ion-mediated toxicity in subsequent biological assays. Thus, toxicity increases over time as nanoparticles aggregate and release additional ions. Moreover, *Daphnia magna* is a non-selective filter feeder that commonly ingests various particles ranging from 0.1 µm to over 50 µm with a high efficiency for particles less than 8 µm (e.g., micro- and nanoplastics)^74^ and metal nanoparticles from suspensions, which allows them to take up particles typically ranging from 20 nm to 64^75^. *Daphnia magna* can internalize and accumulate small nanoparticles, particularly in the digestive tract leading to absorption and oxidative stress, which causes DNA damage. Larger particles hinder physiological functions and cause stress by physically blocking or accumulating in certain tissues, potentially leading to immobility or death^76^ (Supplementary Material, Fig. S9). Nanoparticles may also adsorb onto the carapace surface^71,72^. On the other hand, in addition to the metal NPs’ toxicity, RhB showed moderate acute toxicity in zebrafish (96 h) and *Daphnia magna* (48 h), with studies reporting LC_50_ of 18 and 24 mg/L, respectively^77^. These findings support the hypothesis that only low-dilution members were not associated with effects on daphnia immobility. The toxicity observed in the irradiated, treated supernatant samples was likely attributable to the presence of nanometal particles and released metal ions rather than to RhB, given its initial concentration (4.7 mg/L) and the photodegradation process, which produced no harmful degradation products.

In zebrafish, the embryonic chorion plays a crucial role in maintaining a favorable microenvironment for embryonic development and serves as an effective protective barrier against external contaminants including nanoparticles^78,79^. Structurally, the chorion is characterized by numerous pores with diameters reaching approximately 500 nm^80^, which facilitate, among other functions, the diffusion of oxygen required for normal development. Previous studies have demonstrated a strong affinity between embryonic chorions and nanoparticles, which tend to progressively accumulate on the chorion surface and obstruct these pores^81,82^. Over time, extensive particle deposition may substantially reduce oxygen availability resulting in localized hypoxic conditions that can impair development or increase embryonic mortality. Under standard conditions, zebrafish embryos typically hatch within 48–72 h post fertilization. Following hatching, exposure pathways become more diverse as larvae are no longer protected by the chorion. From this stage onward, particles may interact with the entire body surface, and during the free-feeding phase (96–120 hpf), they may also be ingested^82,83^. After hatching, nanoparticles have been detected at increasing levels in several larval tissues and organs, including the gills, vasculature, digestive system and brain, potentially leading to additional morphological abnormalities and functional impairments^81,82,84^. These mechanisms may collectively account for the observed alterations in mortality values during experimental assessments.

The reduced sensitivity of *A. fischeri* to metal nanoparticles can be attributed to the nature of the Microtox® assay, which primarily reflects short-term metabolic inhibition and is strongly influenced by the bioavailability of dissolved metal ions. Previous studies have shown that ZnO and CuO nanoparticle toxicity in *A. fischeri* is frequently driven by dissolved metal ions. For our result that CuO was non-toxic to *A. fischeri* in the tested range the possible cause is that CuO dissolution is highly conditional and can be suppressed by aggregation and complexation. In some studies, CuO NP toxicity to *A. fischeri* is clearly observed and largely attributed to soluble Cu ions, but effect levels vary substantially with test format and medium^85^. A practical interpretation is that, under the assay conditions (including salinity adjustment typical for Microtox), CuO may have had limited bioavailable Cu^2^⁺ release over the acute exposure window.

Mechanistically, many studies conclude that acute effects of ZnO (and often CuO) in bacterial assays are dominated by dissolution (Zn^2^⁺/Cu^2^⁺) with particle-specific contributions depending on medium chemistry (pH, ionic strength, complexing ligands) and exposure time^86^. Our findings that filtered/supernatant fractions became less toxic in *A. fischeri* (EC₅₀ values shifting above 100% of the original sample) strongly supports the theory that the main drivers of toxicity in the suspensions were particle-associated (e.g., residual particulate matter, surface reactivity, or colloid-bound species). This aligns with work showing that dissolution and speciation govern Microtox® metal responses and that removing particulate phases can markedly reduce apparent toxicity^87^.

Overall, the results confirm that *A. fischeri* represents a comparatively conservative indicator of metal-oxide nanoparticle toxicity and underscore the importance of combining bacterial and whole-organism assays for a comprehensive assessment of nanoparticle-related ecological risks.

## Conclusions

This study demonstrates that ZnO–CuO nanocomposites prepared by a simple mechanical mixing of commercial metal oxide nanopowders can combine high photocatalytic efficiency with an acceptable environmental safety profile. Structural, morphological, and optical analyses of ZnO–CuO nanocomposites confirmed the coexistence of ZnO and CuO and the formation of effective interfacial contacts, which play a key role in charge separation under visible light irradiation. Among the composite samples with various mass ratios between the two metal oxides, ZnO97%–CuO3% NPs exhibited the highest photocatalytic performance toward Rhodamine B degradation representing an optimal balance between heterojunction-driven charge separation and light absorption without excessive surface blocking or recombination losses observed at higher CuO loadings.

Dissolution experiments revealed that Zn^2^⁺ release dominates the ionic contribution of the nanocomposite while Cu^2^⁺ dissolution remains negligible and the fact that metal ion availability is strongly governed by the chemical composition of the aqueous matrix. These findings underline the importance of considering realistic water chemistries when interpreting nanoparticle-related toxicity.

Biological investigations demonstrated that neither the nanocomposite itself nor the photocatalytically treated water adversely affected plant physiological processes, including photosynthesis and respiration in *Ocimum basilicum L.* In contrast, untreated dye-containing water caused severe phytotoxicity. Complementary ecotoxicological assessments across multiple trophic levels confirmed organism-dependent sensitivity, with particle-associated effects playing a major role in acute toxicity while filtered supernatants showed markedly reduced biological impact.

Overall, this integrated evaluation highlights that photocatalytic efficiency alone is insufficient to assess the sustainability of water treatment technologies. By combining material characterization, photocatalytic performance, dissolution behavior and multi-trophic biological assessment, the present work provides a comprehensive framework for the development of environmentally compatible photocatalytic systems. Thus, metal oxide nanocomposites obtained by mechanically mixing technique from commercially available metal oxides emerges as a cost-effective and scalable candidate for sustainable water purification applications.

## Supplementary Information

Supplementary Information.

## Data Availability

The data that support the findings of this study are available from the corresponding authors, (I.Z), (M.E.B.P), upon reasonable request.
