# Supplementary material for: ZnO–CuO nanocomposites for sustainable water treatment linking photocatalytic efficiency with ecotoxicological safety
Source: Sci Rep. 2026 May 25;16:23835. doi: 10.1038/s41598-026-53364-z (PMC13434802; doi:10.1038/s41598-026-53364-z)
Supplement: Supplementary file 1 [file 41598_2026_53364_MOESM1_ESM.docx]

ZnO–CuO Nanocomposites for Sustainable Water Treatment Linking Photocatalytic Efficiency with Ecotoxicological Safety

Irina Zgura^1*†^, Monica Enculescu^1^, Nicoleta Preda^1^, Liviu Nedelcu^1^, Oana Rasoga^1^, Valentin-Adrian Maraloiu^1^, Catalin Negrila^1^, Madalina Cercel^1^, Monica Dinescu^1^, Bogdan Ciprian Mitrea^2^, Tom Matei Iacob^2^, Cornelia Diac^2^, Bogdan Bita^3,4^, Cornelia Nichita^2,5^, Judit Háhn^6†^, Zsolt Csenki-Bakos^6^, Gergő Tóth^6^, Balázs Göbölös^6^, Illés Bock^6^, Florian Littorie^7†^, Pascale Gillon^7^, Marcela-Elisabeta Barbinta-Patrascu^3*†^

^1^ National Institute of Materials Physics, Atomistilor 405A, 077125 Magurele, Romania, [irina.zgura@infim.ro](mailto:irina.zgura@infim.ro), [mdatcu@infim.ro](mailto:mdatcu@infim.ro), [nicol@infim.ro](mailto:nicol@infim.ro), [nedelcu@infim.ro](mailto:nedelcu@infim.ro), [oana.rasoga@infim.ro](mailto:oana.rasoga@infim.ro), [maraloiu@infim.ro](mailto:maraloiu@infim.ro), [catalin.negrila@infim.ro](mailto:catalin.negrila@infim.ro), [madalina.chivu@infim.ro](mailto:madalina.chivu@infim.ro), [monica.daescu@infim.ro](mailto:monica.daescu@infim.ro)

^2^ University of Bucharest, ICUB. Faculty of Physics, CTT-3Nano-SAE Research Centre, MG-38, 405 Atomistilor Street, 077125, Magurele, Romania, [bogdan.mitrea@3nanosae.org](mailto:bogdan.mitrea@3nanosae.org), [tom.iacob@3nanosae.org](mailto:tom.iacob@3nanosae.org), [cornelia.diac@3nanosae.org](mailto:cornelia.diac@3nanosae.org), [cornelia.nichita@unibuc.ro](mailto:cornelia.nichita@unibuc.ro)

^3^ Department of Electricity, Solid-State Physics and Biophysics, Faculty of Physics, University of Bucharest, 405 Atomistilor, 077125 Magurele, Romania, [bogdan.bita@fizica.unibuc.ro](mailto:bogdan.bita@fizica.unibuc.ro), [marcela.barbinta@unibuc.ro](mailto:marcela.barbinta@unibuc.ro)

^4^ Optospintronics Department, National Institute of Research and Development for Optoelectronics-INOE 2000, 409 Atomistilor, 077125 Magurele, Romania, [bogdan.bita@fizica.unibuc.ro](mailto:bogdan.bita@fizica.unibuc.ro)

^5^ National Institute for Chemical – Pharmaceutical Research and Development, 112 Vitan Avenue, 031299, Bucharest, Romania, [cornelia.nichita@unibuc.ro](mailto:cornelia.nichita@unibuc.ro)

^6^ Hungarian University of Agriculture and Life Sciences, Institute of Aquaculture and Environmental Safety, Páter Károly u. 1., H-2100 Gödöllő, Hungary, [Hahn.Judit@uni-mate.hu](mailto:Hahn.Judit@uni-mate.hu), [csenki-bakos.zsolt.imre@uni-mate.hu](mailto:csenki-bakos.zsolt.imre@uni-mate.hu), [toth.gergo@uni-mate.hu](mailto:toth.gergo@uni-mate.hu), [gobolos.balazs@uni-mate.hu](mailto:gobolos.balazs@uni-mate.hu), bock.illes@uni-mate.hu

^7^ GEPEA, UMR 6144, F-44600 Saint Nazaire, France, CNRS, Nantes Université, Oniris, IMT Atlantique, [florian.littorie@univ-nantes.fr](mailto:florian.littorie@univ-nantes.fr), [pascale.gillon@univ-nantes.fr](mailto:pascale.gillon@univ-nantes.fr)

^†^ These authors contributed equally to this work

Correspondence: [irina.zgura@infim.ro](mailto:irina.zgura@infim.ro) (I.Z); [marcela.barbinta@unibuc.ro](mailto:marcela.barbinta@unibuc.ro) (M.E.B.P)

a)

b)

c)

d)

**Figure S1**. EDX mapping and corresponding spectra of metal oxide nanocomposites: a) ZnO99%-CuO1% NPs; b) ZnO97%-CuO3% NPs; c) ZnO95%-CuO5% NPs; d) ZnO90%-CuO10% NPs.

|  |  |
| --- | --- |
| ZnO100%NPs | ZnO99%-CuO1%NPs |
|  |  |
| ZnO97%-CuO3%NPs | ZnO95%-CuO5%NPs |
|  |  |
| ZnO90%-CuO10%NPs | CuO100%NPs |

**Figure S2**. Size distribution of particle population for metal oxide samples based on ZnO, CuO and ZnO-CuO nanocomposites.

a)	b)

c)

**Figure S3**. Survey spectra of ZnONPs sample (a), CuONPs sample (b) and of ZnO97%-CuO3%NPs sample (c).

**Table S1**. Surface composition by XPS measurements (% atomic)

| Sample index | C | O | Zn | Cu |
| --- | --- | --- | --- | --- |
| ZnONPs | 23 | 34.7 | 4.3 | - |
| CuONPs | 24.7 | 34.3 | - | 41 |
| ZnO97%-CuO3%NPs | 28.9 | 32.4 | 37.8 | 0.9 |

(a)	(b)

(c)

**Figure S4**. XPS spectra of C1s for ZnO(a) CuO(b) and ZnO-CuO(c) samples

The peaks A are associated with C-C and C-H bonds, while peaks B indicate the presence of C-O bonds. At the higher binding energies, peaks C may indicate the presence of -COOR or even carbonate groups [51].

(a)	(b)

(c)

**Figure S5**. XPS spectra of O1s for ZnO(a), CuO(b) and ZnO-CuO(c) samples

The O1s spectra also show similarities across all three samples. The peaks A are associated with metallic oxide. The binding energy in the case of ZnO is found to be 530.26eV while in the case of CuO is 529.66eV. The values correspond to those commonly found in the literature [50, 52]. The peaks B are usually associated with C=O double bonds but the formation of metal-O-C or metal-OH bonds is also very likely. In Cu hydroxide O1s has a lower binding energy than in Zn hydroxide, which is also visible in our spectra. The peaks C are assigned to C-O simple bonds but we cannot exclude also the formation of traces of metal carbonates [51].

a)	b)

**Figure S6**. XPS spectra of Zn_LMM Auger from pristine ZnONPs sample (a) and Cu_LMM Auger from pristine CuONPs sample (b).

**Figure S7**. PL spectra of ZnO and ZnO97%–CuO3% nanocomposite (λ=350 nm).

a)	b)

**Figure S8**. Photographs of plant before (a) and after (b) treatment with 10⁻⁵ M RhB solution.

|  |  |  |  |
| --- | --- | --- | --- |
| 0.1 mg/L ZnO97%–CuO3% NPs | 10 mg/L ZnO97%–CuO3% NPs | 100 mg/L ZnO97%–CuO3% NPs | 1000 mg/L ZnO97%–CuO3% NPs |
|  |  |  |  |
| 100 mg/L ZnO97%–CuO3% NPs | 1000 mg/L ZnO97%–CuO3% NPs | 1000 mg/L ZnO97%–CuO3% NPs |  |

**Figure S9**. Representative optical microscopy images illustrate particle accumulation at different exposure concentrations within the digestive tract of exposed organisms.

References

[50] Frankcombe, T. J. Liu, Y. Interpretation of Oxygen 1s X-ray Photoelectron Spectroscopy of ZnO, *Chem. Mater*., **35**, 5468–5474, (2023).

[51] Naumkin, A. V. Kraut-Vass, A. Gaarenstroom, S. W. Powell, C. J. NIST Standard Reference Database Number 20, National Institute of Standards and Technology, Gaithersburg MD, 20899 (2000).

[52] Biesinger, M. C. et al., Advanced analysis of copper X-ray photoelectron spectra, *Surf. Interface Anal*., **49**: 1325–1334 (2017).
